# The Relation of the Laboratory to the Practice of Medicine

**Published:** 1917-07

**Authors:** 


### THE RELATION OF THE LABORATORY TO THE PRACTICE OF MEDICINE.

Although for hundred of years the laboratory has been used to asssist in the diagnosis of disease, the advent, during the past few years, of new and manifestly important methods makes a paper on this subject both important and opportune. The findings and conclusions of the men of research in the laboratories have furnished the key and explanation for some of the most serious diseases from which man suffers. The physician, working under modern methods, can approximate quite accurately the possibility of reviving the function of diseased organs and the outcome or prognosis in a large percentage of his cases. However, there remain many instances in which the end is not certain, and in which the possibilities of recovery are not entirely understood. It is to the research laboratory that we must look for the answer to these very complex and only partially solved problems. The only way in which it can accomplish this result is through cooperation with the physician and surgeon in practice. Progressive physicians regard the laboratory as indispensable and as a prerequisite in the scientific treatment of disease.

It is a significant and fortunate circumstance that increased attention is being directed to the establishment of free laboratories by state and city boards of health. Moreover, most modern hospitals maintain a pathologist who, by means of a well equipped laboratry, is an indispensable aid to attending physicians in diagnosing and estimating the prognosis of disease. The second function of the laboratory which may be of more value, is the search for the solution of unexplained problems. It is obvious, therefore, that the laboratory has come to stay.

Although I realize the great importance of the laboratory in the practice of medicine, I am not of the belief that a man may make every diagnosis in the laboratory. The function of the laboratory is not to diagnose diseases; its task is to report the results to the practitioner, who should use them only as an aid for his own diagnosis. The clinician should also remember that there is only a certain amount of finality in the average laboratory report. Should there be a lack of agreement between the clinical findings and those of the laboratory, the practitioner should always adhere to the former, until there is perfect accord between the two. The sooner he arrives at a point of wholesome skepticism and accepts a laboratory report as of only secondary importance to his clinical findings, the better his results will be.

In sending speciments to the laboratory, practitioners are sometimes disappointed when the report is contrary to what they anticipate. It is this paradox I wish to make clear, and to do this I shall give illustrations from some of the more common diseases in which unexpected reports are often returned.

**Syphilis.**—The three ways in which this disease may be diagnosed in the laboratory are: a miscroscopic examination of the exudate from the chancre, a Wassermann test of the blood serum, and a Wassermann test of the spinal fluid. A positive test in either is more likely to be correct that a negative finding. Since a positive Wassermann is rarely obtained until the disease has progressed for thirty days, the only test which is reliable during the first month is a microscopic examination of the exudate from the chancre. However, if two or three slides have been sent to the pathologist with negative results the patient should be kept under observation for five or six weeks, having a Wassermann done every six or seven days after the third week. If the condition should be one of syphilis, a positive report should be obtained not later than the sixth week. Many persons with syphilis come in for treatment six or eight years after the primary infection, and in these instances a negative blood Wassermann often results. It is in such cases that an examination of the spinal fluid should be made. After repeated negative laboratory reports antileutic treatment is rarely warranted.

**Typhoid Fever.**—Because of its widespread prevalence and serious nature, typhoid fever has been a fruitful field for research. Numerous methods have been introduced as a means of diagnosing this disease. Blood culture and the Widal reaction have been considered by a majority of laboratory men as the most applicable of all.

Blood Culture.—During the first week of typhoid fever, this is by far the best method of diagnosis. However, this is a very difficult test to make unless the blood is collected by a pathologist of experience. I do not recommend it as a routine practice in the country.

Widal Reaction.—This reaction is based on the observation that serum from typhoid fever patients will cause agglutination of the specific bacilli when diluted to the required degree. As a whole this is a very reliable method. Nevertheless, there are certain objections which the clinician must consider. The reaction is seldom obtained before the seventh day of the disease, and in certain cases perhaps not until the convalescent stage. Furthermore, a positive Widal reaction in any person who has received a prophylactic dose of typhoid vaccine of a person who has ever suffered from the disease is of no value in the diagnosis of typhoid fever. Such persons under normal conditions almost invariably give a slight positive reaction, but rarely develop typhoid fever. In suspicious cases, specimens of the blood should be sent to the laboratory every third day until a positive report is obtained or until it is certain that the condition is not one of typhoid fever.

**Malaria.**—It has been proved beyond controversy that malaria is caused by a parasite whih is transmitted from man to man by mosquitoes belonging to the genus Anopheles. This parasite is found in the blood of patients suffering from malaria, in or on the red cells.

The essential factors in the diagnosis of this disease by the laboratory are; tendency to hypoleukocytosis; presence of the malarial parasite in the blood; and absence of a septic factor.

If the patient has not received treatment and the proper search is made, the specific parasite can generally be demonstrated in the blood in all cases of malaria. The best time to collect the blood for examination in the tertian and quartian varieties is at the beginning of or during the paroxysm, while in the estivo-autumnal ten or twenty hours after the paroxysm seems to be the correct time. Obviously, several specimens collected at different hours during the day should be sent to the laboratory.

The following facts should be remembered when sending specimens for examination for typhoid or malaria: the impossibility of examining a single preparation for typhod and malaria; importance of obtaining blood under sterile conditions from the lobe of the ear; necessity in the Widal test of collecting on filter paper two or three drops of blood; and for malaria the necessity of collecting a small drop of blood on a miscroscopic slide, this drop to be spread in a thin, even smear with a second slide.

**Diphtheria.**—In recent years, the diagnosis of diphtheria has been largely made by means of the laboratory. Every clinician should be supplied with sterile test tubes and swabs, and when a suspiious case appears he should make a swab and send it to the laboratory at once. A positive laboratory diagnosis of this condition, the same as in others, is far more reliable than a negative, since the diseased portion of the throat may not be accessible and one's technic in collecting the specimen not always infallible. Since it require about twenty-four hours to obtain a report from a pathologist after the swab has been received at the laboratory, I advise in a very suspicious case, the giving of anti-toxin immediately after the swab has been made.

**Sputum.**—In the examination of sputum the most important pathogenic bacteria which the average physician wishes to know about are the tubercle bacilli. The failure of the pathologist to find such bacteria in a specimen should not cause the physician to believe his diagnosis to be wrong if the clinical symptoms indicate the disease. As a matter of fact, the tubercle bacilli is rarely found until the disease has advanced almost to the point of cavitation.

**Nephritis.**—From our present increased knowledge of pathologic conditions doubtless no disorder, with the exception of syphilis, has profited more than nephritis. The results obtained have been instrumental in affording us a valuable method, not only for diagnosing the existence of the disease, but also in giving us a valuable guide to the treatment and an index to the prognosis of the case.

As an indication of renal insufficiency the clinician for years has depended on the routine examination of the urine. The urine then was examined as to amount, specific gravity, whether it did or did not contain albumin or casts. These reports were anomalous and likely to be confused often with conditions which were not as serious. It has since been shown that a person may pass albumin and casts for years and show practically no pathologic change in the kidneys.

The test which has recently enabled the clinician to diagnose quite acurately the efficiency of the kidneys is the phenolsulphonephthalein test of Rowntree and Geraghty. This test is based on the fact that a normal kidney has the power of eliminating a fairly constant percentage of this dye in a given length of time. The efficiency of the kidney is estimated by the amount of this dye eliminated in a given period. This method furnishes remarkable results as to renal activity, if an examination of the blood is made in connection with it.

The test is performed as follows: thirty minutes before the test is begun the patient receives 400 to 500 c.c. of water; this is given in order that good diuresis may be secured. The patient next receives hypodermically into the deltoid muscle 6 mg. of phenolsulphonephthalein. The patient is then instructed to empty his bladder at once. This may be done by means of a catheter if there shoud be any obstruction. The urine is collected for a period of two hours and ten minutes in two receptacles; the first receptacle to contain the urine passed the first hour and ten minutes; the second receptacle, the urine passed the second period. The ten minutes is added to the first hour because it takes approximately that long for the dye to appear in the urine.

It has been noted that the healthy individual will eliminate 40 to 50 per cent. the first hour, and 20 to 25 per cent. the second hour. In diseased conditions of the renal epithelium the eliminating power of the kidney will be much decreased.

**Conclusion.**—From what has been said above, it is apparent that the research laboratory may increase the efficiency of the average practitioner almost to the point of finality. However, this, as in many other branches of science, depends on the thoughtful cooperation of the physician and the laboratory men concerned.—Journal of Missouri State Medical Association, St. Louis, Mo.

